# Genetic Variation in Morphology, Seed Quality and Self-(in)Compatibility among the Inbred Lines Developed from a Population Variety in Outcrossing Yellow Mustard (*Sinapis alba*)

**DOI:** 10.3390/plants1010016

**Published:** 2012-08-23

**Authors:** Bifang Cheng, David J. Williams, Yan Zhang

**Affiliations:** 1 Agriculture and Agri-Food Canada, Saskatoon Research Centre, 107 Science Place, Saskatoon, SK, S7N 0X2, Canada; Email: david.williams@agr.gc.ca; 2 Mustard 21 Canada Inc., Saskatoon, SK, S7H 5H9, Canada; Email: yan.zhang@agr.gc.ca

**Keywords:** condiment, yellow mustard (*Sinapis alba*), inbreeding, genetic variation, self-(in)compatibility, erucic acid, linolenic acid, mucilage

## Abstract

Yellow mustard (*Sinapis alba* L.) has been grown as an important source of condiment for the spice trade in the world. It is an obligate outcrossing species due to its sporophytic self-incompatibility (SI). To utilize heterosis for yield potential, we have attempted to develop elite component inbred lines for producing high-yielding synthetic varieties for this crop. The open-pollinated variety Andante was used as the initial population. To circumvent the SI barrier, bud-pollination for selfing was performed on the selected initial (S0) plants. Various types of inbreeding depression were observed in the S1 generation. Elite inbred lines tolerant to inbreeding were produced by purging the deleterious alleles in each inbred generation. Self-compatible (SC) lines were developed for the first time in this species. There were three types of erucic variants (high: 49.9%, median: 23.9% and low: 1.4%), three types of linolenic variants (high: 18.5%, median: 13.8% and low: 3.8%) and two types of mucliage variants (high: 164.0 cS*mL/g and low: 12.0 cS*mL/g) among the developed inbred lines. These variants are being used to investigate the genetic and molecular mechanism underpinning the phenotypic variation of the seed oil profile and SI/SC traits in yellow mustard.

## 1. Introduction

Yellow mustard (*Sinapis alba* L.) has been grown as an important source of condiment for the spice trade in the world. The western provinces Manitoba, Saskatchewan and Alberta in Canada are the predominant production areas of this crop. It is more heat and drought tolerant and exhibits better resistance to pod shattering and diseases such as blackleg compared to *Brassica napus* and *B. rapa* [1,2,3]. However, yellow mustard has much lower seed yield compared with canola *B. napus*, brown and oriental condiment mustard *B. juncea*. Therefore, the major breeding objective for yellow mustard has been to increase the yield potential [4].

Yellow mustard is an obligate outcrossing species due to its sporophytic self-incompatibility. Olsson (1960) [5] measured the degree of outcrossing in the field plots of yellow mustard to be as high as 99.6%. Therefore, recurrent selection has been used as the major method for cultivar development and germplasm enhancement in this crop [6,7,8]). Three varieties AC Pennant, AC Base and Andante were developed from the same base population comprising 47 different accessions through several cycles of recurrent selection [8]). Quality traits such as protein and mucilage contents as well as seed size were effectively improved by a number of recurrent selection cycles. However, seed yield was not improved by recurrent selection due to its low heritability.

Heterosis breeding has proven to be a very successful approach for significant increase of seed yield in many crops. Heterosis for seed yield and number of siliques per plant in the hybrids between inbred lines was demonstrated in yellow mustard [9]. However, the development of hybrid cultivars is not feasible in this species due to the following two reasons. Firstly, the self-incompatible reproductive system in yellow mustard makes it difficult to develop and maintain inbred lines for use as parents in hybrid breeding. Secondly, there is no truly functional hybrid system available yet in this crop. Therefore, utilizing (partial) heterosis in synthetic varieties is an alternative approach for increasing the seed yield in yellow mustard in the short term.

The method of bud-pollination for selfing can be used as an approach to circumventing the self-incompatibility barrier for developing elite inbred lines. Various degrees of inbreeding depression will appear among the inbred lines due to the occurrence of homozygosity of deleterious recessive alleles. The elite inbred lines tolerant to inbreeding will be identified and used as components for making high yielding synthetic varieties. In the present paper, we report on the development of diverse elite inbred lines. These inbred lines exhibit great variation in morphology, seed oil profile, and mucilage content. It is of particular interest that we have obtained some self-compatible inbred lines in yellow mustard.

## 2. Results and Discussion

### 2.1. Inbreeding Depression in the S1 Generation

**Figure 1:**
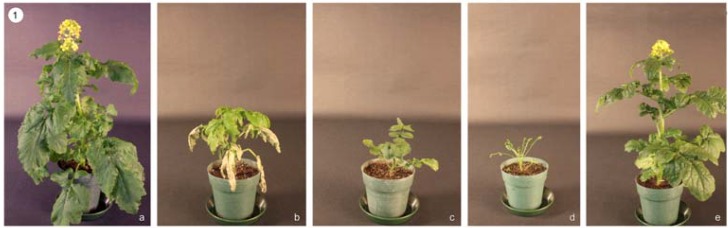
Open-pollinated (S0) and S1 plants of cv. Andante. (**a**) Open-pollinated (S0) plant; (**b**) Abnormal S1 plant with withering leaves; (**c**) Abnormal S1 plant without main inflorescence; (**d**) Weak S1 plant exhibiting reduced leaf size and stunted growth; (**e**) Normal S1 plant.

**Figure 2:**
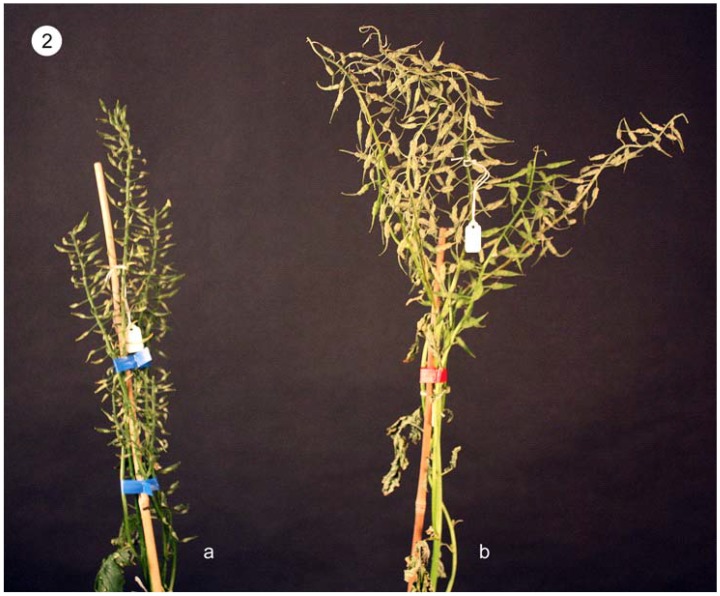
Seed setting of self-incompatible and self-compatible S1 plants. (**a**) Self-incompatible S1 plant; (**b**) Self-compatible S1 plant.

**Figure 3:**
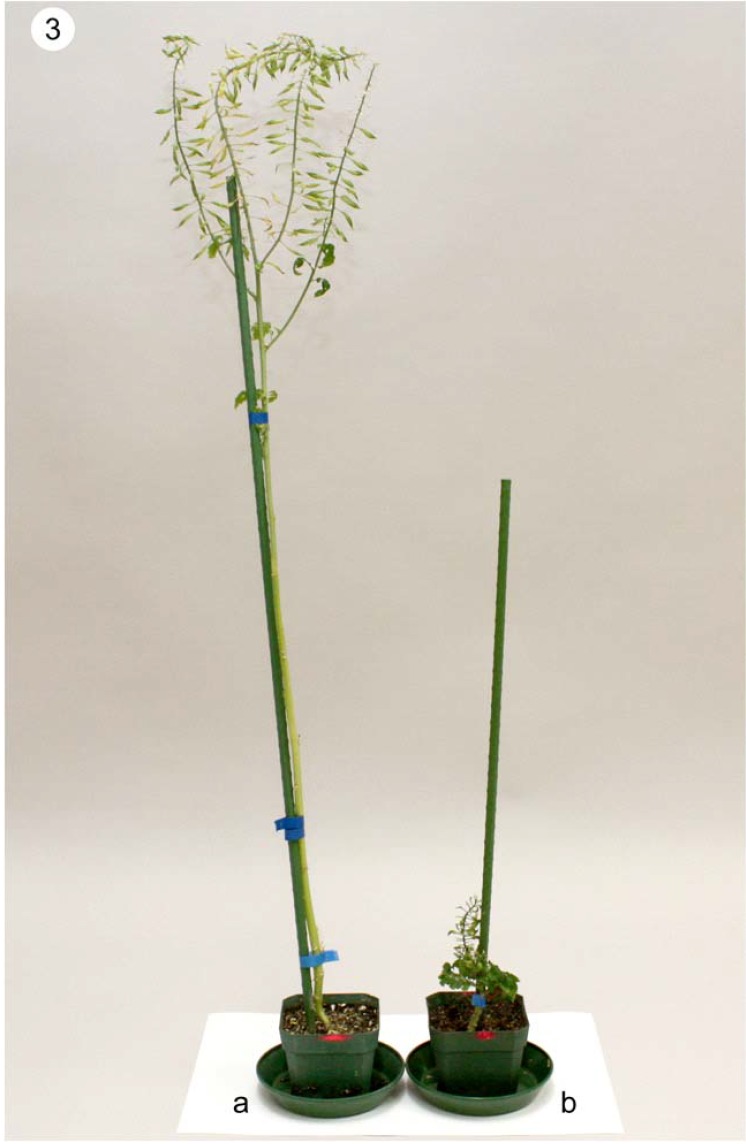
Normal and dwarf S2 plants of line Y665. (**a**) Normal S2 plant; (**b**) Dwarf S2 plant.

The open-pollinated (S0) plants of cv. Andante exhibited great variation in growth vigour, plant height, and flowering time. The weak S0 plants were discarded. The strong S0 plants were selected, of which the main inflorescences were bagged for selfing while the branches were bud-pollinated to circumvent the SI barrier for producing selfed seeds (S1). Thirty plants of each S1 progeny were grown. The S1 plants showed various types and degrees of inbreeding depression as well as great variation in morphology, self-(in)compatibility and fatty acid profile. The S1 plants were classified into four groups based on the inbreeding depression: (1) The abnormal type: Plants had withering leaves (Figure 1b) or had no main inflorescence (Figure 1c); (2) The weak type: Plants exhibited reduced leaf size and stunted growth (Figure 1d); (3) The albino type: The seedlings had whitish cotyledons and died at early stage. (4) The normal type (Figure 1e): Plants were comparable to the open-pollinated (S0) plants (Figure 1a) in morphology and growth vigor. The abnormal and weak S1 plants were discarded. The S1 plants with normal morphology and strong growth vigor were further self-pollinated to produce S2 seeds. From the S2 to S4 generations, strong plants were continuously selected and bagged to produce selfed seeds.

Different S1 progenies exhibited great variation in the extent of inbreeding depression. Out of the 10 S1 progenies studied, four had a high percentage (73.3% to 93.3%) of normal plants; four had 50% to 67.7% normal S1 plants; one S1 progeny had only 24.7% normal plants and the remaining one exhibited severe inbreeding depression with all the S1 plants showing stunted growth and reduced leaf size. Breeding efforts were focused on developing inbred lines tolerant to inbreeding depression from the S1 progenies with a high percentage of normal plants.

Self-pollination of the open-pollinated (S0) plants of yellow mustard resulted in the occurrence of abnormal, weak or albino plants in addition to normal plants in the S1 generation. The observed inbreeding depression in yellow mustard could be attributed to the occurrence of homozygosity of recessive or partly recessive deleterious alleles in the inbred progenies [10,11]. Andante is an open pollinated population variety. In plants with heterozygous loci, the recessive or deleterious recessive alleles were not expressed in the phenotype due to the masking effects by the favourable dominant alleles. Inbreeding of the heterozygous plants has revealed the deleterious recessive traits. Varying degrees of inbreeding depression appeared between different S1 progenies, suggesting that those initial S0 plants carried different proportions of favorable dominant and deleterious recessive alleles. With our objective to develop elite inbred lines, the homozygotes harbouring the favourable dominant alleles were strong and normal, and further used to develop elite inbred lines whereas those undesirable deleterious recessive homozygotes were discarded. Consequently, the frequencies of the favourable dominant alleles were increased in the selected inbred lines. The genetically enhanced diverse inbred lines are valuable components for compositing high-yielding synthetic varieties in yellow mustard.

### 2.2. Development of Self-Compatible Inbred Lines

The open-pollinated plants (S0) of cv. Andante exhibited great variation in the extent of self-incompatibility. Out of the forty S0 plants studied, five plants were found to have a very high self-compatibility Index (SCI) (2 to 4 seeds/pod), ten were partially self-compatible with a SCI of 1 to 2 seeds/pod, the remaining 25 plants had a low SCI (fewer than 1 seed/pod). Figure 2 shows the seedset of a self-incompatible plant (Figure 2a) and a self-compatible (SC) plant (Figure 2b). The S1 progenies from the 15 S0 plants with a SCI of over 1.0 seed(s)/pod were planted. The self-compatible S1 plants were identified in the two S1 progenies Y020 and Y041. Out of the 28 S1 plants in Y020, two were self-incompatible (SCI: <1.0); sixteen plants were self-compatible (SCI: 2–4seeds/pod) and one plant Y020-11 exhibited a very high SCI (6.4 seeds/pod). The remaining nine plants were partial self-compatible. Out of the 19 S1 plants in Y041, four (Y041-1, Y041-3, Y041-13 and Y041-26) were found to be self-compatible (SCI: 2.9–4.8 seeds/pod); and the remaining plants were partial self-compatible (SCI: 1–2 seeds/pod). The five self-compatible S1 plants Y020-11, Y041-1, Y041-3, Y041-13 and Y041-26 were bagged to produce self-pollinated S2 seeds. The S2 plants with strong self-compatibilty were further self-pollinated for 3 cycles to S5 and selection was performed in each generation for strong self-compatibility and growth vigour. As a result, ten self-compatible S5 inbred lines tolerant to inbreeding depression have been developed from the five self-compatible S1 plants. The S1 plants with partial self-compatibility were further self-pollinated for 2–3 cycles to develop partial self-(in)compatible inbred lines. In each generation, efforts were directed to select the strong and partial self-compatible plants for further inbreeding. So far sixty-five elite partial SC inbred lines have been produced.

Fan *et al*. [12] reported the occurrence of variation in the extent of self-(in)compatibility among different cultivars in yellow mustard. However, further effort has not been made to exploit this variation to develop genetically stable self-compatible breeding lines. In our studies, plants with self-compatibility allele(s) were identified in the S1 generation and used to develop genetically stable SC lines via pedigree breeding. Identification of the self-compatible trait has a significant impact on the breeding strategy for yellow mustard. It will enable the breeding method to switch from heterogeneous population improvement to homozygous line variety development. Little is known about the inheritance of self-(in)compatibility in yellow mustard. The SC lines produced in the present study will be used to study the inheritance of this trait and develop diagnostic molecular markers (e.g., intron length polymorphisms, ILPs) for the SC trait for further marker assisted breeding applications. Furthermore, the SC trait of the SC lines can be transferred into different elite self-incompatible or partial self-compatible inbred lines to develop superior SC pure-line varieties with high seed yield potential and improved quality profile. Such SC lines can be used as parents for hybrid development once a hybrid system becomes available in this crop.

### 2.3. Variation of Morphological and Seed Quality Traits between Different Inbred Lines

Different S2 or S3 inbred lines were developed from the various open-pollinated S0 plants of cv. Andante. The inbred lines had great variation in morphology, plant height, and flowering time. Some inbred lines had leaves with fewer lobes and dark green colour, while others had leaves with more lobes and light green colour. These inbred lines can be easily differentiated from one another based on their leaf morphology. Plant height of different inbred lines varied from 55 cm to 150 cm. A dwarf variant with a height of only 5.5 cm (Figure 3b) was observed in the S2 line Y665.

Different S2 lines also exhibited variation in erucic acid content. The S2 plants with 40.0–55.0% erucic acid content were stable and no segregation in erucic content was observed in the following generations. However, the S2 plant Y061 containing an erucic acid content of 38.8% continued to segregate in the S3 generation. Out of the 14 S3 plants from Y061, eight had an erucic acid content of 42.6% to 49.4%, which was stable in the following inbred generations; two plants with an erucic acid content of 36.5–37.3% continued to segregate in the S4 generation; the remaining four S3 plants with an erucic acid content of 23.9% to 27.5% were fixed in the subsequent inbreeding generations. Half-seed analysis of the line W96-1-2 identified plants with erucic acid content ranging from 0.9% to 6.5% Successive inbreeding of the plants with low erucic acid content (range: 0.9–1.7%) resulted in the production of inbred lines with truly low erucic acid content (1.4%). Based on the erucic acid content, inbred plants (lines) of yellow mustard can be divided into three types: (1) High erucic acid type: Inbred lines such as Y661 had a high erucic acid content (49.9%) in the seed; (2) Median erucic acid type: Inbred lines such as Y496 produced 23.9% erucic acid of total fatty acids in its seed. (3) Low erucic acid type: Inbred line (Y1130) contained 1.4% erucic acid in the seed.

Most of the S2 and S3 inbred plants/lines contained a linolenic acid content of 10–13%. For instance, the S2 line Y061 had a linolenic acid content of 13.8%. All nine S3 lines derived from Y061 had similar linolenic level (range: 9.3–13.2%), indicating that this trait was genetically fixed. However, low as well as high linolenic variants were identified in different S2/S3 progenies. The S2 line Y158 had a low linolenic acid content (3.8%) and was self-pollinated to produce S3 seeds. All nine S3 lines from Y158 had very low linolenic acid level (range: 2.2–3.4%), which were stable in the following S4 and S5 generations. The S3 line Y240 had a high level of linolenic acid (18.5%) in the seed. The S4 lines derived from Y240 continued to contain a high linolenic acid (17.2–20.8%) similar to the S3 line Y240.

The cv. Andante had a mucilage content of 46.2 cS*mL/g seed [8]. However, different inbred lines developed from Andante ranged in mucilage content from 12.0 cS*mL/g seed to as high as 164.0 cS*mL/g seed. The high mucilage (>100 cS*mL/g seed) or low mucilage (<20 cS*mL/g) lines identified in the S3 generation were stable in the S4 generation, suggesting that they were homozygous for the respective high or low alleles controlling the mucilage content. The inbred lines with the intermediate mucilage content of 50–60 cS*mL/g did not breed true and continued to segregate into low or high content in the subsequent generations.

Yellow mustard germplasm with different erucic acid contents was reported before [4,13,14,15]. Zero erucic acid (0.1%) line was developed via inbreeding at Agriculture and Agri-Food Canada-Saskatoon Research Centre (AAFC-SRC) [16,17]. The inheriance of erucic acid content was studied by crossing the high erucic acid variety Sabre (>50%) with the zero erucic acid line W96-2 (<0.1%), which revealed that this trait was controlled by a single gene exhibiting partial dominance of high over low content [18]). In the present study, we have isolated three types of erucic acid variants via successive inbreeding of the heterozygous open-pollinated plants. The high erucic variant (inbred line Y661) had a high erucic acid content (49.9%). The intermediate erucic variant (inbred line Y496) and the low erucic variant (inbred line Y1130) were new and had an erucic acid content of 23.9% and 1.4%, respectively. Genetic studies on the erucic acid content of the available four variants WD96-2 (0.1%), Y1130 (1.4%), Y496 (23.9%), Y661 (49.9%) are underway and will reveal whether the four levels of erucic acid content are controlled by multiple alleles at a single locus or by different gene loci in yellow mustard. Moreover, the cloning and sequence comparison of the erucic gene from the four variants will be performed in order to reveal the molecular mechanism underpinning the variation of erucic acid content in yellow mustard.

Traditional *Brassica* species (*B. napus*, *B. rapa* and *B. juncea*) oilseed cultivars contained 9% linolenic acid in their seed oil [19]. The low linolenic acid gene source in *B. napus* was produced by seed chemical mutagenesis treatment of a high linolenic Canadian canola *B. napus* cv. Oro [20]. Interestingly, inbreeding of the open-pollinated plants of cv. Andante in yellow mustard resulted in isolation of three types of linolenic acid variants. Lines Y240, Y061 and Y158 had high (18.5%), median (13.8%) and low (3.8%) linolenic acid contents, respectively. These three linolenic variants will be used to study the inheritance of linolenic acid content and to further characterize the molecular basis underlying the variation of this trait in yellow mustard. In addition, the low linolenic acid line Y158 will be crossed with the canola-quality yellow mustard line (low erucic acid and low glucosinolate contents) produced at AAFC-SRC to develop a canola yellow mustard line with similar fatty acid profile (high oleic and low linolenic acid) to canola *B. napus*.

Mucilage of yellow mustard seed contributes to the consistency of the prepared mustard products [21] and is therefore a very important seed quality parameter for the condiment mustard processors. Variation of mucilage content has been reported in yellow mustard [22,23]. Mucilage is located in the epidermal layer of the testa in yellow mustard [24] and *B. campestris* [25]. Studies on inheritance of mucilage content in *B.* *campestris* indicated that mucilage in the seed coat was determined by the genotype of the maternal plant and controlled by two genes exhibiting dominance epistasis [25]. Undestanding the inhertance of mucialge content in yellow mustard is very important for designing breeding strategy to develop cultivars with different mucilage contents for the condiment mustard industry. The inbred lines with low and high mucilage contents will be used to study the inheritance of mucilage content in this crop.

Open-pollinated population varieties of yellow mustard comprise great genetic variation. Molecular marker such as AFLP (amplified fragment length polymorphism) analysis revealed the partitioning of total variation into 79.1% within accession and merely 20.9% between accessions [26]. In the present study, the observation of various types of morphological and quality-profile variants has provided further evidence for the vast genetic variation harboured within the existing open-pollinated population cultivars such as Andante in yellow mustard.

## 3. Experimental Section

The open-pollinated population variety Andante of yellow mustard was used as the initial population for inbred line development in the present study. Andante was developed from the base population comprising 47 accessions through several cycles of recurrent selection for improved quality traits at AAFC-SRC [8]. Andante has a higher mucilage content (46.2 cS*mL/g seed) and larger seed size compared with other cultivars (AC Pennant and AC Base). The yellow mustard line W96-1-2 with an erucic acid content of 1–5% produced at AAFC-SRC was used as gene source to develop inbred lines with low erucic acid content.

### 3.1. Self-(in)Compatibility Measurement

Forty open-pollinated plants (S0) of cv. Andante were grown in the greenhouse. The main inflorescence of the strong S0 plants were bagged for self-pollination and the branches were bud-pollinated to produce the selfed S1 seeds. The seed setting of the first 20 pods on the main inflorescence were used to calculate the self-compatibility index (SCI) for each plant. The SCI is calculated as follows: SCI = Number of self-pollinated seeds/number of self-pollinated pods. The S0 plants were classified into three types according to the SCI: (1) The self-incompatible (SI) plants have a SCI lower than 1.0; (2) The partially self-compatible plants have a SCI of 1.0–2.0; and (3) The self-compatible (SC) plants with a SCI higher than 2.0. The SC plants were used to develop SC inbred lines via pedigree breeding.

### 3.2. Fatty Acid Profile of Seed Oil and Mucilage Content Analysis

A bulk of ten selfed seeds of each inbred line was analyzed for fatty acid profile. The seed fatty acid profile was determined following the method described by Raney *et al*. [16]. For mucilage content analysis, one hundred plants of each inbred line were grown and open-pollinated in the field in 2011. The open-pollinated seeds of each inbred line were harvested in bulk and used as seed sources for the analysis of mucilage content. The mucilage content of the samples was calculated as the viscosity of extract minus the viscosity of water multiplied by the volume of water divided by the weight of the seed (cS*mL/g seed) [23].

## 4. Conclusions

Elite self-compatible and partial self-compatible inbred lines tolerant to inbreeding have been developed in yellow mustard. The genetically enhanced diverse lines are valuable parental components for producing high yielding synthetic varieties for this crop. Different inbred lines exhibit great variation in morphology, the extent of self-(in)compatibility, fatty acid profile and mucilage content. The availability of these inbred lines will make it possible to investigate the genetic and molecular mechanism underpinning the phenotypic variation of these traits in yellow mustard.
